# Bacteriology

**Published:** 1904-07-02

**Authors:** 


### BACTERIOLOGY.

**Bacteriology of Empyema in Children.**—Bythell[1] has made an examination of forty cases of empyema in children. The pus was drawn off by means of a sterile needle, films stained with methylene blue, Gram and Ziehl Neelsen, and cultures made on agar or blood agar. The organisms found were Fränkel's pneumococcus, Friedlander's pneumo-bacillus, Staphylococcus albus, S. aureus, S. cereus albus, streptococcus, Micrococcus tetragenus, tubercle bacillus, and one unidentified bacillus. Pneumococci he distinguishes from streptococci by their lanceolate shape, presence of capsules, absence of growth on gelatin in forty-eight hours, and by the absence of isolated round forms; tubercle bacilli were discovered in films from two of the cases.

**Acute Rheumatism.**—Beattie[2] confirms the work of Poynton and Paine in regard to the etiology of rheumatic fever. He made cultures in milk broth from the knee-joints of a fatal case of acute rheumatism and endocarditis in a woman; the diplococcus rheumaticus was found associated with putrefactive organisms. In a second fatal case of rheumatism and endocarditis in a young girl there was slight patchy congestion of both knee-joints, and cultures showed the specific diplococcus which, when inoculated into rabbits, gave rise to endocarditis, chorea, and arthritis, and from these lesions the diplococcus was recovered. The cardiac vegetation proved on section to contain large masses of the diplococcus, the synovial membranes were thickened and somewhat gelatinous in appearance. Beattie favours the theory that the diplococcus is the specific organism of this disease. He concludes that Achalme's bacillus must have been an accidental contamination, and notes that Dana, who confirmed Achalme's work, states that in two of his first five cases a diplococcus was found associated with the bacillus. Poynton,[3] in a contribution on the infective nature of rheumatic fever, gives details of the bacteriological findings in a case of acute rheumatism with endocarditis which subsequently developed a fatal malignant endocarditis. Pure cultures of the diplococcus rheumaticus were found in tubes inoculated from the valves, the infarcated spleen and the kidney. The diplococcus was found with other organisms in the pneumonic lungs. The pericardium and blood gave negative results. Inoculated upon monkeys and rabbits, arthritis, endocarditis and pericarditis were produced. He points out that as the cocci are carried in the blood stream they will first of all be found in the connective tissues around the blood-vessels, and it will only be in the later stages that they can be detected in the blood, synovial fluid, or pericardial effusion. The establishment of the infective theory of acute rheumatism lends support to the existence of such lesions as true rheumatic broncho-pneumonia, pleurisy, nephritis, and peritonitis. The case cited is a striking proof that simple and malignant endocarditis can be stages of the same process.

**Trypanosomiasis.**—This subject has attracted considerable attention during the past six months. In January an article appeared by Dutton, Todd, and Christy,[4] dealing with human trypanosomiasis on the Congo. They state that only one type of trypanosome has been met with in human blood, whether there were signs of sleeping sickness or not. In the cerebro-spinal fluid other forms may possibly be found. The type in the blood has always been T. Gambiense, and in inoculated rats this is the only form developed, whether the material be taken from a case of sleeping sickness or of simple trypanosomiasis, and whether blood or cerebro-spinal fluid be used. They presume, therefore, that the disease on the Congo is caused by the Gambian parasite either in its long form with long flagella or in its stumpy form with short flagella. The discussion on Trypanosomiasis before the Epidemiological Society brought out the general agreement amongst experts that the disease was transmitted by means of a fly, and that this fly was the Glossina palpalis. There was a difference of opinion as to whether the parasite went through any developmental stage in the body of the tsetse fly, and no theory was advanced as to how the parasite excited the symptoms of sleeping sickness. Mott suggested that Castellani's diplococccus, which is also associated with this disease, played a subsidiary part only as a secondary infection. Louis Sambon[5] maintains that the tsetse fly is a true alternative host, not a mere passive carrier of the disease. He describes asexual forms of trypanosome which multiply by longitudinal division, sexual forms, large, stumpy, macrogametes, with short flagellum, and small, hyaline, slender, microgametes, with large, deeply-staining nucleus, large micronucleus, and long flagellum. He suggests that sections of infected tsetse flies might show these forms, and instances the striking resemblance to the halteridium seen in birds. Adams,[6] of the Gambia Protectorate, who was one of the first to suggest that trypanosomiasis in Europeans and sleeping sickness in negroes were identical diseases, states that the tsetse fly is not found in the Gambia settlement, although sleeping sickness is not uncommon. He inclines to the view that the intermediate host is a rat, and that man is infected by the bite of this animal. He mentions, as an alternative, a fly of the species Tabanidæ, found in the mangrove swamps. Should it be found that the life history of the trypanosome gambiense resembles that of other trypanosomes, the course would be somewhat as follows:—A tsetse fly biting a man, horse, or ox infected with trypanosomes would become itself infected. The trypanosomes multiply in the stomach of the fly, pass into the blood-vessels, and become non-mobile and perhaps intra-corpuscular. The male and female forms are less resistant than the neuters, and these wander into the blood and lymph spaces, and are carried to the pharynx and ovaries of the fly. From the pharynx they pass to the proboscis, and when the fly bites they infect again man or cattle. The forms in the ovaries infect the next generation of flies.

[1] Jour. of Path. and Bact., March, 1904. [2] Ibid. [3] Brit. Med. Jour., May 14, 1904. [4] Ibid., Jan. 23, 1904. [5] Ibid., March 19, 1904. [6] Ibid., April 16, 1904.

